# A Minimal Model Shows that a Positive Feedback Loop Between sNHE and SLO3 can Control Mouse Sperm Capacitation

**DOI:** 10.3389/fcell.2022.835594

**Published:** 2022-03-25

**Authors:** Bertrand de Prelle, Pascale Lybaert, David Gall

**Affiliations:** Research Laboratory on Human Reproduction, Faculté de Médecine, Université libre de Bruxelles, Brussels, Belgium

**Keywords:** sperm, capacitation, SNHE, SLO3, hyperpolarization, mathematical model, bistability

## Abstract

When mammalian spermatozoa are released in the female reproductive tract, they are incapable of fertilizing the oocyte. They need a prolonged exposure to the alkaline medium of the female genital tract before their flagellum gets hyperactivated and the acrosome reaction can take place, allowing the sperm to interact with the oocyte. Ionic fluxes across the sperm membrane are involved in two essential aspects of capacitation: the increase in intracellular pH and the membrane hyperpolarization. In particular, it has been shown that the SLO3 potassium channel and the sNHE sodium-proton exchanger, two sperm-specific transmembrane proteins, are necessary for the capacitation process to occur. As the SLO3 channel is activated by an increase in intracellular pH and sNHE is activated by hyperpolarization, they act together as a positive feedback system. Mathematical modeling provides a unique tool to capture the essence of a molecular mechanism and can be used to derive insight from the existing data. We have therefore developed a theoretical model formalizing the positive feedback loop between SLO3 and sHNE in mouse epididymal sperm to see if this non-linear interaction can provide the core mechanism explaining the existence of uncapacited and capacitated states. We show that the proposed model can fully explain the switch between the uncapacitated and capacited states and also predicts the existence of a bistable behaviour. Furthermore, our model indicates that SLO3 inhibition, above a certain threshold, can be effective to completely abolish capacitation.

## 1 Introduction

Despite continuous research in reproductive biology over the last two decades, the prevalence of couple infertility (over 12 months) remains around 15%, among which 30% is due to a male infertility factor (Hajder et al., 2016). The sperm count has been continuously decreasing for 40 years, raising the alarm for a major fertility crisis by the midst of the 21st century and the need for increased research in male infertility (Barratt et al., 2017; Levine et al., 2017; Duffy et al., 2020). Our knowledge of the molecular regulation of sperm motility and its fertilization potential is still incomplete and the etiology of a number of human male infertility cases remains unknown. Therefore, whether in search of new male fertility screening methods or novel contraceptive solutions, a deeper understanding of the molecular events regulating sperm functions is needed. These functions notably depend on ion homeostasis, which is controlled by ion channels and transporters. Many of these proteins or their regulatory subunits are expressed exclusively in sperm cells, making them ideal pharmacological targets (Wang et al., 2021).

Before mammalian spermatozoa are able to fertilize the oocyte, they need to spend some time in the female genital tract. In human this duration must be of several hours while in mouse it is around an hour. During this transit, spermatozoa are exposed to a diversity of environmental and intracellular signals allowing sperm to acquire a special form of motility, known as hyperactivation, and the ability to undergo the acrosome reaction. This process is called capacitation and since its discovery (Austin, 1951; Chang, 1951), *in vitro* studies of mammalian sperm showed that the presence of albumin and bicarbonate in the physiological incubation medium is essential for capacitation to occur (Lee and Storey, 1986; Stival et al., 2015).

Mammalian sperm capacitation is characterized by an increase in intracellular pH (pH_i_) (Zeng et al., 1996), membrane hyperpolarization (Arnoult et al., 1999) and a calcium influx from the extracellular medium (Ruknudin and Silver, 1990), and since nearly three decades now, pharmacological and genetic studies have revealed the presence of sperm-specific proteins that are essential for male fertility and necessary for the sperm to reach capacitation: The sNHE sodium-proton exchanger, the SLO3 potassium channel and the CATSPER calcium channel. The absence of any of the corresponding genes leads to male infertility without any systemic abnormality, in accordance with the fact that these proteins are expressed only in the sperm (Ren et al., 2001; Wang et al., 2003; Santi et al., 2010).

The calcium influx, increase in pH_i_ and hyperpolarization observed during capacitation are dependent on the activity of CATSPER, sNHE and SLO3 (Carlson et al., 2003; Wang et al., 2003; Santi et al., 2010), and these actors operate all together as CATSPER and SLO3 are activated by an elevation of pH_i_ and sNHE is activated by hyperpolarization (Schreiber et al., 1998; Kirichok et al., 2006; Windler et al., 2018). These considerations led Chávez et al. (2014) to the proposal of the existence of a positive feedback loop between the activation of sNHE and SLO3, leading to a high pH_i_ and membrane hyperpolarization, that could promote the pH_i_-dependent CATSPER’s activity during capacitation.

In order to test this hypothesis and find if capacitation can indeed be controlled by the feedback loop between sNHE and SLO3, we propose a mathematical model for the capacitation process based on these two essential molecular actors of capacitation. The model also includes the known effect of PKA-dependent phosphorylation on SLO3, and even though the capacitation process includes other regulatory changes, the focus is here set on a minimal number of actors that could be at the core of a capacitation switch. Using this minimal model we then investigate the conditions necessary for the incubation medium to promote capacitation and ask whether this process of capacitation can be reversed back. Finally we also investigate the most effective way of preventing capacitation by inhibition of sNHE and SLO3.

## 2 Materials and Methods

Our model is a minimal two variables model describing the evolution in time of the intracellular pH (pH_i_) and the transmembrane electrical potential (V_m_) of a mouse epididymal spermatozoon, which includes the feedback between the increase in pH_i_ and hyperpolarization resulting from the activations of sNHE and SLO3.

**Table:** 

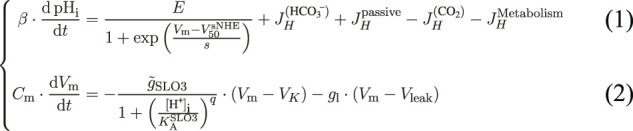

**FIGURE 1:**
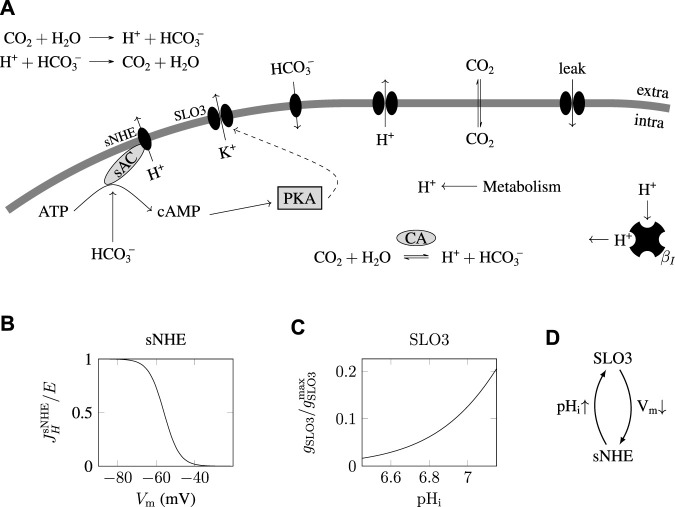
The feedback loop regulating capacitation. **(A)** The model takes into account the potassium channel SLO3 and the sodium-proton exchanger sNHE. SLO3 is activated downstream of cAMP production through the activation of PKA. The cAMP production by the soluble adenylyl cyclase is activated by the presence of bicarbonate (HCO_3_^−^). A leak current, regrouping all transmembrane electrical currents except through SLO3, is taken into account. HCO_3_^−^ passes through the plasma membrane by a variety of transporters. The carbon dioxyde (CO_2_) freely and instantly crosses the cell membrane, and equilibrates with HCO_3_^−^ and H^+^. An influx of protons resulting from the metabolism is taken into account and the intracellular pH varies due to a leak of protons through the plasma membrane. Finally the total intrinsic protons buffer *β*_*I*_ is present. **(B)** sNHE activation curve against V_m_. Hyperpolarization activates sNHE. In this graph, the intracellular cAMP concentration, which influences the half activation voltage, is fixed at 1 mM. The *y*-axis label denominator *E* is the maximal activity of sNHE. **(C)** SLO3 activation against pH_i_. Intracellular alkalization activates SLO3. In this graph the transmembrane potential V_m_ is fixed at +80 mV and the phosphorylation level *P*_SLO3_ is kept at 0 (unincubated spermatozoa). The *y*-axis label denominator $g_{\text{SLO}3}^{\text{max}}$ denotes ${\tilde{g}}_{\text{SLO}3}$ in the text. **(D)** An increase in pH_i_ activates SLO3 which lets potassium ions flow out and so hyperpolarizes the cell. This hyperpolarization activates sNHE which in turn extrudes protons and so increases pH_i_.

The first Eq. 1 links the evolution of pH_i_ to the different mechanisms related to proton homeostasis (Figure 1A), where the *β* factor is the total pH_i_ buffer capacity of the sperm cell. The second Eq. 2 is based on the conservation of the electrical charge and shows that the evolution of the transmembrane potential depends on the contributions of SLO3 and a leak current. The factor C_m_ is the membrane’s capacitance of the sperm cell. All the actors taken into account in the two equations of the model are schematized on Figure 1A.

The first term on the right side of Eq. 1 represents the contribution of the sperm-specific sodium-proton exchanger sNHE. This contribution is always positive as the extrusion of protons has the effect of increasing the pH_i_. This transporter has a putative voltage sensor (Wang et al., 2003) and is activated by hyperpolarization in sea urchin sperm (Windler et al., 2018) (Figure 1B). Moreover, the half maximal activation’s voltage of sNHE, $V_{50}^{\text{sNHE}}$ , depends on the intracellular cAMP concentration. As the cAMP_i_ production by the soluble adenylyl cyclase (sAC) is a function of the concentration of HCO_3_^−^, $V_{50}^{\text{sNHE}}$ can be modeled as a Hill function as follow:

$$V_{50}^{\text{sNHE}}={\tilde{V}}_{50}^{\text{sNHE}}+k_{\text{sNHE}}\cdot \frac{1}{1+{\left(\frac{K_{Ab}^{\text{sNHE}}}{{\left[{HCO}_{3}^{\;-}\right]}_{i}}\right)}^{2}}$$ (3)

The constant ${\tilde{V}}_{50}^{\text{sNHE}}$ is the half maximal activation’s voltage of sNHE in absence of bicarbonate. The maximal activation shift of sNHE, *k*_sNHE_, has been set to the value of 14 mV, corresponding to experimental data where [cAMP]_i_ = 1mM, which is well above the physiological level (Garbers et al., 1982; Jansen et al., 2015). Precise data for the voltage activation parameters of sNHE against [HCO_3_^−^]_i_ is not available in the literature; yet, based on the percentage of hyperpolarized sperm against the bicarbonate concentrations obtained by Escoffier et al. (2015) showing a jump around or below 7 mM, it is reasonable to take $K_{Ab}^{\text{sNHE}}$ at 3 mM.

In our model, the soluble adenylyl cyclase activation is controlled directly by the intracellular bicarbonate concentration. Intracellular calcium concentration is also known to activate this adenylyl cyclase (Litvin et al., 2003). As a first approximation, our model indirectly includes this effect of sAC’s activation by calcium through $K_{Ab}^{\text{sNHE}}$ because an elevation of calcium is concomitant with an elevation of bicarbonate. Experimental data shows indeed that the addition of bicarbonate to the sperm incubating medium leads to a 1 minute scale increase in pH_i_ (which is concomitant with a [HCO_3_^−^]_i_ increase by virtue of equation 11) and [Ca^2+^]_i_ (Luque et al., 2018; Chávez et al., 2019). Finally, the pH_i_’s dependence of the activity of sNHE is taken into account as follow:

$$E=\frac{E_{\text{sNHE}}}{1+{\left(\frac{K_{A}^{\text{sNHE}}}{{\left[H^{+}\right]}_{i}}\right)}^{j}}$$ (4)

The maximal activity of sNHE (*E*_sNHE_), $K_{A}^{\text{sNHE}}$ and *j*, for which data is not yet available for mouse sperm, have been set to the values obtained for sodium-protons’ exchangers in fibroblasts (Boron, 2004). All parameters are given in Supplementary Appendix Table S1.

The other contributions to the variation in pH_i_ of the sperm cell are the following:

Firstly the contribution from the flux of bicarbonates, $J_{H}^{\left({{HCO}_{3}}^{\;-}\right)}$ , representing the sum of the fluxes through all the bicarbonate transporters. To date, the bicarbonate transporters reported in sperm are SLC26A3 (Wang et al., 2021), SLC26A6 and a putative electroneutral anion exchanger (Chávez et al., 2012), possibly a Na^+^/HCO_3_^−^ cotransporter (Demarco et al., 2003; Vyklicka and Lishko, 2020), and the cystic fibrosis transmembrane regulator (CFTR) (Hernández-González et al., 2007; Xu et al., 2007; Escoffier et al., 2012). This bicarbonate flux, including both active and passive transports, is reduced to its most simple expression of a linear dependence on the transmembrane gradient of the bicarbonate concentration $\left({\left[{HCO}_{3}^{\;-}\right]}_{e}-{\left[{HCO}_{3}^{\;-}\right]}_{i}\right)$ , as shown in Eq. S1 in Supplementary Appendix. We made this choice as we are building a minimal model of sperm capacitation; only the core process of the feedback loop, comprising SLO3 and sNHE, is treated to produce the significant shifts in V_m_ and pH_i_ of the cell, regardless of details in the bicarbonate transports.

Secondly, a term of passive flux of protons across the membrane $J_{H}^{\text{passive}}$ , described by the Goldman-Hodgkin-Katz equation for electrodiffusion (Eq. S2 in Supplementary Appendix), for which the permeability is here chosen in the physiological range (Putnam, 2012). This term was added to the model in order to prevent a shift to extra-low pH_i_ values when [HCO_3_^−^]_e_ is abruptly changed.

The next contribution to the variations in pH_i_ comes from the effect of changes in extracellular carbon dioxide concentration which are instantly followed by the same intracellular carbon dioxide concentration changes as the permeability of the plasma membrane to CO_2_ is high. So in the model, the CO_2_ concentration is the same on both sides of the sperm membrane and an increase CO_2_ results in acidification of the cell as the CO_2_ is converted to bicarbonates and protons. This intracellular equilibration between carbon dioxide and bicarbonate is fast as the carbonic anhydrases [CAII, Wandernoth et al. (2015)] catalyse the reversible reaction, as schematized in Figure 1A. In the extracellular medium, as the incubation medium is devoid of carbonic anhydrases as it is usually the case *in vitro* incubations, the equilibration should be considered not instantaneous, but for simplicity we do not include this delay in our model. We nevertheless carried out simulations using explicitly the reaction rates of the conversion of CO_2_ into protons and checked that the results and conclusions presented in this paper are not different (data not shown).

The last contribution to the variations in pH_i_ is the acid loading resulting from the metabolism, taken as a constant. This acid loading, which allows the sperm to reach an equilibrium state by compensating the pH_i_ increase due to the protons’ extruders, includes the contribution of the leak of protons from the acrosome which is an acidic organelle that has been shown to alkalize during capacitation (Nakanishi et al., 2001). The value of this parameter is adjusted so that the pH_i_ of the sperm cell in uncapacitating conditions lies in the range of the experimental data of the literature, between 6.4 and 6.85 (Zeng et al., 1996; Carlson et al., 2007; Chávez et al., 2019).

The first term on the right side of Eq. 2 is the contribution of SLO3, considered with its auxiliary subunit LRRC52 (Yang et al., 2011), to the transmembrane electrical potential. It is activated by alkalization as shown by the Hill’s [H^+^]_i_ dependence of its conductance. The H^+^_i_ concentration for half occupation of SLO3 (written $K_{\text{A}}^{\text{SLO}3}$ ) and the cooperativity coefficient *q* of this Hill’s function have been determined by Yang et al. (2011) using heterologous expression of SLO3 and LRRC52 in oocytes, and the corresponding activation’s curve is drawn on Figure 1C for uncapacitated spermatozoa.

The factor ${\tilde{g}}_{\text{SLO}3}$ appearing in the contribution from SLO3 includes a factor representing the phosphorylation by the cSrc kinase which occurs downstream of the activation of the soluble adenylyl cyclase by intracellular bicarbonate, as follows:

$${\tilde{g}}_{\text{SLO}3}=\tilde{g}\cdot \left(1+\alpha \cdot P_{\text{SLO}3}\right),\;P_{\text{SLO}3}=\frac{1}{1+{\left(\frac{K_{Ab}^{\text{SLO}3}}{{\left[{HCO}_{3}^{\;-}\right]}_{i}}\right)}^{n}}$$ (5)

where P_SLO3_ is the phosphorylation level of SLO3 and is chosen as a Hill equation. The pathway to SLO3 phosphorylation is initiated by the activation of the soluble adenylyl cyclase (sAC) from an increase in [HCO_3_^−^]_i_, which is followed by the activation of the protein kinase A (PKA) and finally leads to the auto-phosphorylation of the cSrc kinase (Stival et al., 2015) which then phosphorylates tyrosine residues of SLO3. In fact, the phosphorylation of SLO3 is slow, of a timescale of 15 min (Stival et al., 2015), but we do not include this delay in our equations because we will focus on the results concerning the steady states of the sperm cell that are reached during incubations in various media. The half maximal concentration $K_{\text{Ab}}^{\text{SLO}3}$ is set at a value around 5 mM as shown in Supplementary Appendix Table S1, and the value of the cooperativity coefficient *n* is set at 6, as explained in Supplementary Appendix. Finally the SLO3’s conductance depends not only on pH_i_ but also on V_m_ (Zeng et al., 2015) and this is taken into account in the factor $\tilde{g}$ appearing in the SLO3’s conductance, independently of the pH_i_ activation, as follows:

$$\tilde{g}=\frac{{\tilde{g}}_{\text{SLO}3}^{\text{max}}}{1+\exp-\left(\frac{V_{\text{m}}-V_{50}^{\text{SLO}3}}{s_{\text{SLO}3}}\right)}$$ (6)

where the Boltzmann activation parameters were measured by Zeng et al. (2015) with electrophysiological techniques on spermatozoa at a pH_i_ of 8. This way of modeling the voltage activation of SLO3 independently of the intracellular pH value is based on experimental activation curves obtained by Yang et al. (2011) for various membrane voltages that indicate a pH_i_-independent voltage activation’s behavior.

The second term on the right of Eq. 2 is regrouping into a leak current the contribution from all other electrogenic transporters of the sperm cell membrane, where the resultant leak conductance *g*_l_ is a constant (the value of which is described in Supplementary Appendix). This choice of taking solely SLO3 as the effector of V_m_ changes during capacitation is due to the fact that we are considering the core model of a feedback loop in order to build a minimal model of capacitation. This choice is supported by the demonstration that the variation in SLO3’s conductance is responsible for the hyperpolarization of the sperm cell during capacitation (Santi et al., 2010; Chávez et al., 2013). The value of V_leak_ is set at −40 mV according to the transmembrane voltage obtained by Chávez et al. (2013) for SLO3’s knock-out and SLO3’s inhibited mouse spermatozoa, both giving a value around −40 mV.

Finally, it could be objected that because the Na^+^/HCO_3_^−^ cotransporter has been suggested to induce hyperpolarization upon addition of bicarbonate (Demarco et al., 2003), the model should include this contribution explicitly. In our approach, this effect was not taken into account as this instantaneous hyperpolarization seems to be transient and disappears in minutes (Demarco et al., 2003; Santi et al., 2010; De La Vega-Beltran et al., 2012; Chávez et al., 2013; Escoffier et al., 2015; Stival et al., 2015). Yet we do not exclude the participation of the cotransporter in the subsequent capacitation-related hyperpolarization as its activity is voltage-dependent. The model could be refined by including such voltage-dependent contributions as the Na^+^/HCO_3_^−^ cotransporter or the CFTR channel among others.

With this minimal model built around the two actors SLO3 and sNHE, we made simulations of the time evolution of the state of a sperm cell in order to check if it gives account of the capacitation process and if a positive feedback between sNHE and SLO3 could be at the core of the process. We also simulated the effects of the inhibition of SLO3 or sNHE on the state of the sperm.

The simulations, using the evolution Eqs 1, 2, were performed using the software XPPAUT 6.11 (Free Software Foundation Inc., Cambridge, United States). In all simulations, the extracellular pH (pH_e_) is fixed at the value of 7.4 and the temperature is set at 37°C, as usually the case in sperm incubating media. Original source code is available on GitHub^1^.

## 3 Results

### 3.1 The Positive Feedback Between SLO3 and sNHE Causes Capacitation

In order to find if the model reproduces the transition from a depolarized acidic state (not promoting capacitation) to a hyperpolarized alkaline state (promoting capacitation, or “capacitating”) of the sperm, we performed a simulation of the incubation of a sperm cell as it is usually done in the laboratory, consisting of the incubation into a solution containing 15 mM bicarbonate ([HCO_3_^−^]_e_ = 15 mM).

**FIGURE 2:**
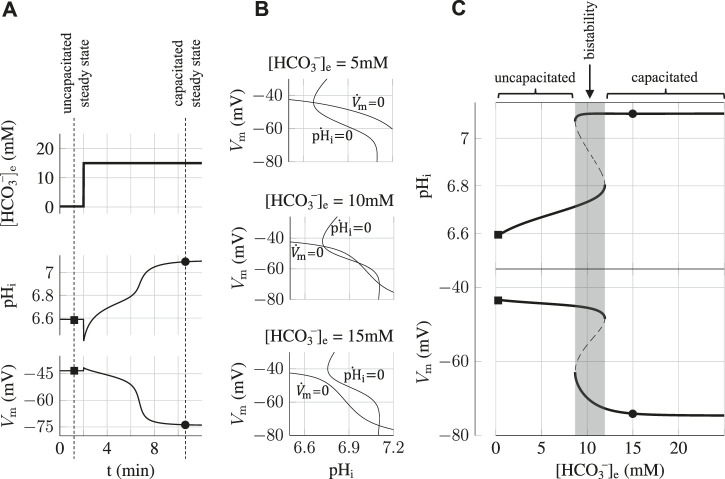
Transition between uncapacitated and capacitated state as the bicarbonate concentration is increased. **(A)** Time evolution of the pH_i_ and V_m_ following a step from 0.2 to 15 mM extracellular bicarbonate $\left({\left[{HCO}_{3}^{\;-}\right]}_{\text{e}}\right)$ at time *t* = 2 min. Following the increase in ${\left[{{HCO}_{3}}^{-}\right]}_{\text{e}}$ , the pH_i_ rises from the initial uncapacitated value of around 6.6 to the capacitating value above 7.1. This significant increase comes with the cell’s hyperpolarization from −45 mV to −75 mV and reflects the feedback loop between SLO3 and sNHE. Beware the instant phosphorylation of SLO3 used in the simulations induces a transition in the 5 min’ time scale, faster than the 15 min timescale necessary for the hyperpolarization of mouse sperm. **(B)** Graphs of the nullclines of the mathematical model for three different extracellular bicarbonate concentrations. Equalling to zero the two Eqs 1, 2 leaves us with two nonlinear equations of the two variables pH_i_ and V_m_, providing two lines (called nullclines) in the (pH_i_,V_m_) plane as shown on each three graphs. The lines labeled $\dot{V_{\text{m}}}=dV_{\text{m}}\;/\;dt=0$ correspond to the states for which the transmembrane potential does not change with time, and the lines labeled $\dot{{pH}_{\text{i}}}=0\;\left(=d\;{pH}_{\text{i}}\;/dt\right)$ correspond to the states for which the cytosolic pH of the sperm does not change with time. The intersections of these two lines give the steady states of the sperm cell at which both V_m_ and pH_i_ do not change in time. The graph on top shows the two nullclines for 5 mM HCO_3_^−^_e_; middle graph: 10 mM HCO_3_^−^_e_; bottom graph: 15 mM HCO_3_^−^_e_. **(C)** Bifurcation diagrams showing the steady states of the sperm as a function of the concentration of ${HCO}_{3\text{e}}^{\;-}$ . The upper diagram shows the pH_i_ of the steady states and the lower diagram shows the transmembrane potential *V*_m_ of the steady states. On the left of the gray region appear in thick solid lines the stable steady states corresponding to an uncapacitating sperm. On the right of the gray region (i.e., for high ${\left[{{HCO}_{3}}^{-}\right]}_{\text{e}}$ ) appear the stable steady states (thick solid line) corresponding the capacitating sperm. The transition between the uncapacitating state and the capacitating state occurs in the gray region where two stable steady states and one unstable steady state (represented in dashed line) are found. This gray region corresponds to the values of [HCO_3_^−^]_e_ where the system is bistable: The sperm can be found in two different stable steady states.

This protocol is represented on the top graph of Figure 2A showing a step of 15 mM of bicarbonate at time *t* = 2 min. The initial concentration of bicarbonate in the medium is set at [HCO_3_^−^]_e_ = 0.2 mM, which corresponds to a solution of pH = 7.4 at equilibrium with ambient atmosphere. As indicated in the graphs of the pH_i_(t) and V_m_(t) on Figure 2A, before the bicarbonate step (t < 2 min) the sperm is at a steady state of (pH_i_,V_m_) ≃(6.6,−45 mV). After the 15 mM pulse of bicarbonate at time *t* = 2 min, the state of the sperm shifts to a pH_i_ of 7.1 and a V_m_ of −75 mV. This transition of the sperm’s state is here fast, of the timescale of 5 min; however, adding the phosphorylation delay of SLO3 results in a transition’s timescale of 15 min (data not shown), in accordance with data of the literature for capacitation of mouse sperm cells (Arnoult et al., 1999; Stival et al., 2015).

This simulation of the time evolution of the state of the sperm therefore shows two steady states, one at 0.2 mM HCO_3_^−^_e_ and one at 15 mM HCO_3_^−^_e_, which are reported on the two diagrams of Figure 2C (black squares and bullets), one corresponding to the uncapacitated state of the sperm and one corresponding to the capacitating state of the sperm.

### 3.2 Bistability of the Capacitation Process

Having found two steady states of the sperm, one at 0.2 mM HCO_3_^−^_e_ and one at 15mM HCO_3_^−^_e_, we wondered if these sperm states were the only possible ones for each of these extracellular bicarbonate concentration, and whether there is a threshold in [HCO_3_^−^]_e_ above which the uncapacitated sperm will spontaneously shift to the capacitating state of low V_m_ and high pH_i_. As a positive feedback loop can give rise to bistability we can expect that for some values of the parameter [HCO_3_^−^]_e_ we would find two possible stable states, one capacitating and one uncapacitating.

The top graph of Figure 2B shows that when [HCO_3_^−^]_e_ = 5 mM there is only one possible steady state for the sperm. When [HCO_3_^−^]_e_ = 15 mM (bottom graph of Figure 2B), there is also only one steady state, at the point (pH_i_,V_m_) ≃(7.1,−75 mV). This latter state is hyperpolarized and alkaline, and so corresponds to a sperm’s capacitating state, as the one found in Figure 2A where [HCO_3_^−^]_e_ = 15 mM.

Between these two bicarbonate concentrations of 5 and 15 mM however, at [HCO_3_^−^]_e_ = 10 mM we find three intersections of the nullclines, which are shown in the middle graph of Figure 2B, and indicating that the sperm can be found in three different steady states, one of these three states corresponding to a capacitating state of the sperm at V_m_ ≃−70 mV and pH_i_ ≃7.1. In order to gain insight of what is happening here we computed the steady states for all values of [HCO_3_^−^]_e_ and plotted these steady states in two diagrams shown on Figure 2C, where we observe that a transition between uncapacitating states and capacitating states happens when the extracellular bicarbonate concentration crosses a threshold at around 12 mM HCO_3_^−^_e_. Below 8 mM HCO_3_^−^_e_ spermatozoa are in uncapacitating states and above 12 mM HCO_3_^−^_e_ are found the capacitating states of the sperm, and between these two appears a region (shaded in the figure) where three steady states of the sperm coexist. The analysis of the dynamics of the system shows that the steady states in the middle (dashed lines in Figure 2C) are unstable; in reality the sperm will never be found at such steady states because any imperceptible fluctuation in the physiological conditions brings the sperm state away from these unstable steady states. Therefore, in the shaded region, the sperm can be found in two different steady states; it is a phenomenon of bistability where the state of the spermatozoa will depend on what happened before they were brought to this bistable region. Indeed, if we start on the left of the diagram where [HCO_3_^−^]_e_ is low, and increase slowly the medium’s bicarbonate concentration to the bistable region, the sperm will be found at the state (pH_i_,V_m_) ≃(6.7,−45 mV), but if the sperm is prepared on the right of the diagram, decreasing the [HCO_3_^−^]_e_ to the gray region will keep the sperm at the capacitating state near (7.1,−70 mV).

### 3.3 The Capacitation Switch has Hysteresis yet is Reversible

The bistability arising from the feedback loop between sNHE and SLO3 implies that the sperm will chose one of the two stable states according to its initial state. This hysteresis phenomenon implies that once the sperm has chosen one of the two stable states, it will remain locked in this state regardless of small changes in the surrounding physiological conditions.

**FIGURE 3:**
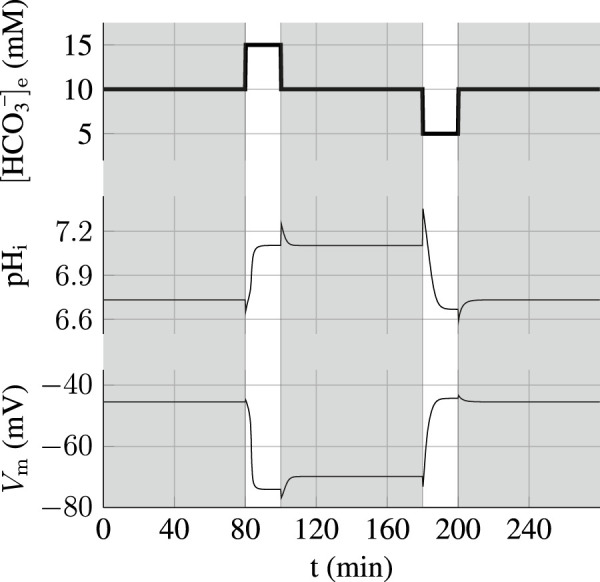
Hysteresis and reversibility of the capacitation process. After having prepared the sperm at 10 mM ${HCO}_{3\text{e}}^{-}$ , a pulse of 15 mM ${HCO}_{3\text{e}}^{-}$ at *t* = 80 min switches the sperm to the capacitating state (i.e., *V*_m_ ≃-70 mV and pH_i_≃7.1). However, when ${HCO}_{3\text{e}}^{\;-}$ is brought back to the initial value of 10mM, the sperm remains in the capacitating state. Nevertheless, when ${HCO}_{3\text{e}}^{\;-}$ is pulsed down to 5 mM for 20 min, the state switches back to the initial non-capacitating state. The extracellular pH is always fixed at 7.4.

That is what we show here with a simulation of an experiment where a hysteresis effect resulting from this bistability arises in the state of the sperm. The simulation, shown on Figure 3, consists in preparing the sperm cell in the bistability region at 10 mM HCO_3_^−^_e_ before imposing a pulse of 15 mM HCO_3_^−^_e_ that brings the sperm to a capacitating state. We find that the capacitating state is robust as the sperm remains in the hyperpolarized high pH_i_ state when the bicarbonate concentration is brought back to its initial value of 10 mM after the pulse. This is a hysteresis effect and it shows that once the sperm cell is in the capacitating state, it is blocked in this state regardless of small variations in the physiological conditions surrounding the sperm cell. We find however that this switch is reversible as a sufficient depletion of [HCO_3_^−^]_e_ for some time is able to bring the sperm back to the uncapacitating state.

We would like to insist here that a capacitating state does not mean a capacitated state, we are rather assessing the intracellular ionic state consistent with sperm incubated in capacitating conditions.

### 3.4 SLO3’s inhibition is More Robust Than sNHE at Preventing Capacitation

**FIGURE 4:**
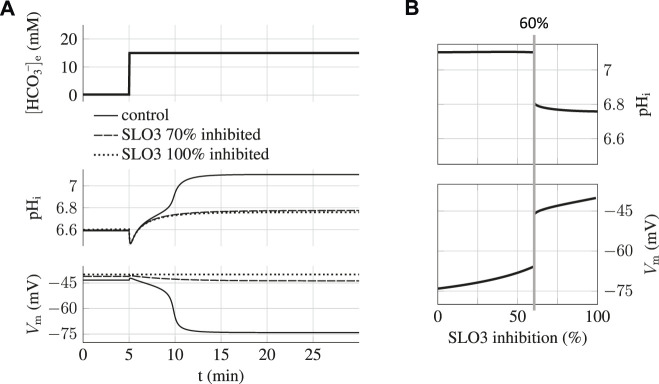
Capacitation dependence on SLO3 inhibition. **(A)** When SLO3 is 70% inhibited, the transition to the capacitated state does not occur and the state is blocked at the values of pH_i_ = 6.8 and V_m_ = −45 mV. In the case of 100% inhibition of SLO3, the model shows no variation in V_m_ during capacitation. **(B)** Graph of the steady states of the sperm against the percentage of SLO3 inhibition. These are the steady states reached after the incubation in capacitating medium (15 mM bicarbonate) of spermatozoa previously prepared at 0.2 mM bicarbonate. A threshold appears at 60% inhibition above which both the increases in pH_i_ and hyperpolarization are much reduced.

The proposed minimal model can also be used as a tool in order to predict how the capacitation process can be blocked efficiently. We simulated the time evolution of the sperm’s states when either SLO3 or sNHE is inhibited and have examined the transition between the uncapacitating and capacitating states. The result of the simulation of the incubation of sperm in 15 mM HCO_3_^−^_e_ when SLO3 is inhibited at a level of 70% is presented on Figure 4A and shows that capacitation can not occur anymore; the sperm does not hyperpolarize and the intracellular pH shows an increase of only 0.2 unit.

By carrying on the same simulations with different inhibition percentages of SLO3 we find a threshold at 60% above which this ions’ fluxes aspect of capacitation is prevented (Figure 4B); the pH_i_ will increase no more than 0.2 unit, from 6.6 to less than 6.8, and the transmembrane potential will not hyperpolarize below −45 mV. The existence of this inhibition threshold above which the sperm does not capacitate anymore is due to the fact that the system is brought into the bistable region and will therefore remain blocked to the uncapacitated state during incubation.

**FIGURE 5:**
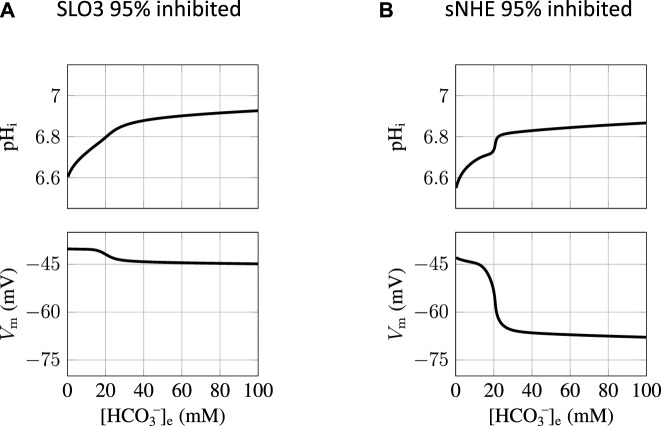
SLO3 inhibition is more robust than sNHE at preventing capacitation. **(A)** When SLO3 is 95% inhibited, the switch to the capacitated sperm does not occur for any HCO_3_^−^_e_ concentration. **(B)** When sNHE is 95% inhibited, the capacitation switch is not prevented when the HCO_3_^−^_e_ concentration exceeds 20 mM.

When simulating an incubation at the same concentration of 15 mM HCO_3_^−^_e_ but with 70% inhibition of sNHE, the effect on capacitation is similar to the SLO3 inhibition (Supplementary Figure S1), but when increasing the HCO_3_^−^_e_ concentration of the incubation medium we find that the sNHE inhibition is not as effective at preventing the capacitation switch as the inhibition of SLO3. This is illustrated on Figure 5 showing the states of incubated spermatozoa as a function of the [HCO_3_^−^]_e_ at a fixed 95% inhibition. The SLO3 inhibition keeps the state depolarized for all bicarbonate concentrations (Figure 5A) but in contrast, when [HCO_3_^−^]_e_ crosses the threshold of 20mM, the capacitation switch is not prevented by a 95% sNHE inhibition (Figure 5B). The prevention of the hyperpolarization in the case of the SLO3’s inhibition is due to the fact that its conductance remains small compared with the leak conductance.

## 4 Discussion

In this work we have built a minimal model for the ions fluxes aspect of capacitation of mouse spermatozoa as a function of the incubating medium’s bicarbonate concentration. We modeled the sperm’s state evolution on the minimal basis of the effect of sNHE and SLO3 on pH_i_ and V_m_. By doing so, for the homeostasis of sperm pH_i_ we included, in addition to sNHE and SLO3, the alkalizing effect of bicarbonate transporters and the acidifying effect of the metabolism of the sperm cell.

The majority of the parameters values used in the model are based on existing data, with the exception of three which remain free. These free parameters are the acid loading from the metabolism and the bicarbonate concentration for downstream half activation of sNHE or SLO3. Nevertheless, the acid loading from the metabolism, which compensates the alkalizing effects of the protons and bicarbonate transporters in order to allow pH homeostasis, is inherently physiological as the alkalizing parameters are physiological.

Concerning the half activation of sNHE and SLO3 downstream of the protein kinase A activation by bicarbonate, our parameter’s values are taken consistently with results from Stival et al. (2015) giving the percentage of hyperpolarized spermatozoa as a function of the HCO_3_^−^_e_ concentration, which indicates a threshold below 7 mM HCO_3_^−^_e_ above which a substantial proportion of spermatozoa do hyperpolarize during the incubation. However, the exact value of these two parameters do not affect the essence of the feedback loop and bistability in our model; it rather shifts the shaded bistability region (Figure 2C) to the right or to the left. In particular, decreasing $K_{\text{Ab}}^{\text{sNHE}}$ will shift the capacitation threshold from around 12 mM (as in Figure 2C) to a lower concentration (data not shown).

Our minimal model gives account of the experimentally observed hyperpolarization and alkalization of the mouse sperm’s cell during capacitation. Indeed the hyperpolarization of the sperm’s cell to −75 mV lies in the range (V_m_<70 mV) found in the literature for capacitated spermatozoa (Arnoult et al., 1999; Escoffier et al., 2015). We emphasize here that our results concern individual spermatozoa that effectively capacitate, and not a “capacitated population” of spermatozoa that consists of capacitated and uncapacitated subpopulations for which the measured hyperpolarization represents the sum of the depolarized uncapacitated subpopulation and the hyperpolarized capacitated subpopulation. In such heterogenous populations, the measured hyperpolarization is around −60 mV (Arnoult et al., 1999; Stival et al., 2015).

Concerning the evolution of the sperm’s pH_i_ during capacitation, our model shows an increase in pH_i_ from 6.6 before incubation to 7.1 after incubation. The uncapacitated value given by the model was in fact adjusted to the value of 6.6 obtained by Chávez et al. (2019), and the capacitated value of 7.1 is in the range around 7.2 obtained recently by Ferreira et al. (2021). However, we would like to stress here that our model’s pH_i_ values of 6.6 before capacitation and 7.1 after capacitation are not fixed and do depend on the exact value of the acid load from the metabolism which is a free parameters as mentioned here above.

Our analysis of the sperm’s steady states as a function of the bicarbonate concentration in the incubating medium showed the existence of a bistability region where the sperm can be found in two different states and we showed that once the sperm has reached the hyperpolarized and alkaline state of capacitated sperm, it will remain capacitated regardless of small fluctuations in the bicarbonate concentration. In addition, we show that this hysteresis phenomenon in the cellular response allows the encoding of transient signals, like a pulse in the external bicarbonate concentration, by long-lasting changes both in pH_i_ and V_m_. This capacitation switch can be reversed back as a sufficiently strong decrease in [HCO_3_^−^]_e_ will bring the sperm back to an uncapacitating state. The existence of this hysteresis phenomenon resulting in a capacitation threshold which is different from the decapacitation threshold is a prediction of the model that can be tested experimentally. On a functional level, the predicted hysteresis, inducing a shift in bicarbonate sensitivity, allows the capacitation process to be more robust in presence of local variations of bicarbonate concentration. This ensures that, once capacitated, sperm cells are less sensitive to these fluctuations, providing a possible explanation for how sperm persist capacitating even when they travel through various parts the female genital tract.

The reversibility of capacitation is still a matter of debate. Even though the reversibility of capacitation could be abolished by other factors not included in the model, such as the degradation of Catsper (Ded et al., 2020), several aspects of capacitation have reversible characteristics. Indeed, various studies indicate that the responses of the sperm state to stimuli such as pulses in bicarbonate concentration, intracellular pH, intracellular calcium concentration, alkaline depolarization, and epididymal extract addition have reversible characteristics (Oliphant and Brackett, 1973; Babcock and Pfeiffer, 1987; Suarez et al., 1987; Espinosa and Darszon, 1995; Schreiber et al., 1998; Wennemuth et al., 2003). We nevertheless insist that the reversibility predicted by the present mathematical model holds for the switch between the acidic depolarized state and the alkaline hyperpolarized state of the sperm, and not for the whole process of capacitation.

Concerningthe inhibition of capacitation, when the sperm is incubated in a 15 mM bicarbonate solution we find a switch at 60% inhibition of SLO3 above which capacitation is prevented. Even though the inhibition of sNHE has similar effect on capacitation in an incubation medium of 15 mM bicarbonate, we find that inhibiting SLO3 is more effective than inhibiting sNHE when the bicarbonate concentration is increased. Indeed, a 95% inhibition of SLO3 prevents capacitation for all possible bicarbonate concentrations that could be found in the genital tract of the mammals (Maas et al., 1977), which is not the case when instead sNHE is 95% inhibited. This stronger effect of SLO3 inhibition on capacitation is not surprising as we may expect inhibition of ion channels to have a much larger impact on membrane potential than the inhibition of a carrier system like sNHE, which has a much lower turnover rate for ion transport. It appears therefore that the inhibition of SLO3 is a better candidate for the development of non-hormonal male contraception. The human sperm however is different from the mouse as hSLO3 is strongly activated by calcium and less by pH (Brenker et al., 2014). Our model could be adapted and applied to human sperm by adjusting the activation curves of SLO3 and taking into account the voltage-gated hydrogen channel 1, HV1, which has been shown to be the dominant proton conductance in human sperm (Lishko et al., 2010). However, as this transporter is not activated by hyperpolarization as it is the case for sNHE, another feedback loop could take place, between hSLO3 and Catsper, as it has been shown that the variations in hyperpolarization and intracellular calcium concentration of human spermatozoa are interconnected during capacitation (Balestrini et al., 2021).

The proposed minimal model has allowed us to identify the core molecular mechanism which is likely to control murine sperm capacitation. It may be extended to include calcium dynamics and further refined to the human case. Concerning the increase in intracellular calcium during capacitation, the feedback loop between sNHE and SLO3, which robustly switches the state of the sperm to an elevated intracellular pH, is likely to keep the pH_i_-sensitive Catsper channel activated during capacitation, and moreover, the reversibility of the switch implies that this capacitation’s aspect of calcium influx can be reversed back. In addition, as in a first approximation we have modeled the soluble adenylyl cyclase activity only through the increase in intracellular bicarbonate concentration and not by explicit increases in intracellular calcium concentration which are known to activate this adenylyl cyclase (Litvin et al., 2003), an extended version of the model, explicitly including Catsper, could take into account this additional feedback. In this field, a first mathematical model of mouse sperm intracellular calcium dynamics has been established by Olson et al. (2010). In their model, they explain the tail to head Ca^2+^ signal propagation following the activation of Catsper, as well as the subsequent sustained calcium increase in the sperm’s head. To address this, they take into account the Ca^2+^ release from the redundant nuclear envelope which was modeled to be indirectly gated by variations in intracellular Ca^2+^ concentration through the production of inositol 1,4,5-trisphosphate from the phospholipase C. Recently, the inclusion of Catsper into a more complex mathematical model of mouse sperm capacitation has been carried out by Aguado-García et al. (2021) in a comprehensive study of early steps of capacitation. Interestingly, this latter study predicts that if capacitation is impaired by *in silico* SLO3’s loss of function, it can not be recovered by overactivation of sNHE. On the contrary, they show that the overactivation of SLO3 can recover the capacitation response of sNHE’s loss of function. This result is now corroborated by our modeling study, highlighting that SLO3 is essential for the capacitation process. Yet we emphasize here again that our model describes the states of the sperm reached after long durations of incubations.

To conclude, using a modeling approach, we have identified a possible core molecular mechanism underlying the transition between the uncapacitated and capacitated state in mouse sperm and studied its dynamic as well as identified pharmacological targets regulating this process. These results may be relevant in the possible development of treatments of male infertility and non-hormonal male contraception, as the inhibition or stimulation of these actors of sperm’s capacitation can modulate the ability of sperm to fertilize the egg. For this, the proposed model should be extended to the human case.

Such a minimal model gives direction for further experimental studies aimed at understanding various aspects of sperm dynamics, and moreover, additional phosphorylation and transport mechanisms of interest could be studied by adding explicitly their contribution to this model.

## Data Availability

Publicly available datasets were analyzed in this study. This data can be found here: https://github.com/bdeprelle/MouseCapaModel.

## Footnotes

1
https://github.com/bdeprelle/MouseCapaModel
