# Supplementary material for: A Minimal Model Shows that a Positive Feedback Loop Between sNHE and SLO3 can Control Mouse Sperm Capacitation
Source: Front Cell Dev Biol. 2022 Mar 25;10:835594. doi: 10.3389/fcell.2022.835594 (PMC8990769; doi:10.3389/fcell.2022.835594)
Supplement: Supplementary file 1 [file Image1.pdf]

frontiers

Supplementary Material

1 SUPPLEMENTARY APPENDIX AND FIGURE

1.1 Appendix

In this section are presented the complete set of functions and equations of the model in addition to the main equations (1) and (2). A table of the values of the parameters used in the simulations of the model is also given.

$$J_H^{(\mathrm{HCO_3^-})} = \frac{k_{\mathrm{bicar}}}{1+\left(\frac{K_\mathrm{A}^{\mathrm{bicar}}}{[\mathrm{H^+}]_\mathrm{i}}\right)^4} \cdot ([\mathrm{HCO_3^-}]_\mathrm{e} - [\mathrm{HCO_3^-}]_\mathrm{i}) \quad \text{(S1)}$$

$$J_H^{\mathrm{passive}} = \frac{1}{\mathrm{V}} \cdot \mathrm{S} \cdot \mathrm{P_H} \cdot \frac{F \cdot V_\mathrm{m}}{RT} \cdot \frac{[\mathrm{H^+}]_\mathrm{i} - [\mathrm{H^+}]_\mathrm{e} \cdot \exp\left(\frac{-F \cdot V_\mathrm{m}}{RT}\right)}{1-\exp\left(\frac{-F \cdot V_\mathrm{m}}{RT}\right)} \quad \text{(S2)}$$

$$J_H^{(\mathrm{CO_2})} = \frac{1}{\beta_I} \cdot \frac{K_\mathrm{a}}{[\mathrm{H^+}]_\mathrm{i}/\mathrm{B_I} + [\mathrm{HCO_3^-}]_\mathrm{i}} \cdot \frac{\mathrm{d}[\mathrm{CO_2}]_\mathrm{e}}{\mathrm{d}t} \quad \text{(S3)}$$

$$\beta = \beta_{\mathrm{CO_2}} + \beta_I = \frac{[\mathrm{HCO_3^-}]_\mathrm{i}}{\log_{10} \mathrm{e}} + \mathrm{B_I} \cdot \frac{[\mathrm{H^+}]_\mathrm{i}}{\log_{10} \mathrm{e}} \quad \text{(S4)}$$

Concerning the bicarbonate dynamics, the following equilibrium equation is used, both for extracellular and intracellular medium concentrations:

$$K_{a1} \cdot K_h \cdot [\mathrm{CO_2}] = [\mathrm{HCO_3^-}] \cdot [\mathrm{H^+}] \quad \text{(S5)}$$

The value of the α factor appearing in equation (5) and representing the relative increase of the SLO3's conductance when phosphorylated, is fixed by assuming that the $\mathrm{pH_i}$ and V_m of the capacitated spermatozoa is 7.1 and -80mV respectively. Indeed, when using these latter values with the results of potassium permeabilities before and after capacitation of sperm populations obtained by Chávez et al. (2013), together with the percentage of spermatozoa that effectively do capacitate in the populations subjected to capacitating conditions (Escoffier et al., 2015) and with the values of the $\mathrm{pH_i}$ before capacitation found in Chávez et al. (2019), our description of SLO3's conductance predicts then $\alpha = 8.3$.
We then set the value of g_l to 1 so that the model accounts for the sperm's transmembrane voltage shift from around -40mV to around -80mV during capacitation (Chávez et al., 2013).
Finally the value of the cooperativity coefficient n for the SLO3 activation by $[\mathrm{HCO_3^-}]_\mathrm{i}$ (equation (5)) has been set at the value of 6 in order for the evolution V_m(t) predicted by the model (including here the phosphorylation delay) to reproduce the results of the time evolution of V_m obtained by Stival et al. (2015) which show a hyperpolarization occurring between 15 minutes and 30 minutes of incubation in a 15mM $\mathrm{HCO_3^-}_\mathrm{e}$ capacitating medium (data not shown).

Other common constants used in the model at the temperature of the simulations T=310K in physiological solutions: V_K=$-84.9\,\mathrm{mV}$, F=$9.65 \times 10^4\,\mathrm{C\,mol^{-1}}$, R=$8.31\,\mathrm{J\,mol^{-1}\,K^{-1}}$, K_{a1}=$2.8 \times 10^{-4}\,\mathrm{M}$ (carbonic acid's first dissociation constant).

Table S1. Parameters values used in the simulations of the model.
The references used in this table are the following: [1]:Windler et al. (2018), [2]:Jansen et al. (2015), [3]:Garbers et al. (1982), [4]:Yang et al. (2011), [5]:Zeng et al. (2015), [6]:Kirichok et al. (2006), [7]:Yeung et al. (2002), [8]:Chávez et al. (2013), [9]:Escoffier et al. (2015), [10]:Magid and Turbeck (1968), [11]:Boron (2004), [12]:Putnam (2012)
* Data obtained for other cell types than mouse spermatozoa, where the values have been adjusted for the sperm's cytosolic volume.

**Parameters**		**Value**	**References**
$J_H^{\mathrm{Metabolism}}$	acid loading from metabolism	$5\,\mu\mathrm{M/L}$	free parameter
$\mathrm{E_{sNHE}}$	sNHE activity (for the whole membrane)	$4\,\mathrm{mM/s}$	[11]*
K_A^{sNHE}, j	Hill parameters for sNHE's $\mathrm{pH_i}$ activation	$0.3\,\mu\mathrm{M}$, 4	[11]*
$\tilde{\mathrm{V}}_{50}^{\mathrm{sNHE}}$, s	sNHE voltage activation parameters	$-70\,\mathrm{mV}$, $4\,\mathrm{mV}$	[1]
$\mathrm{k_{sNHE}}$	sNHE's maximal voltage activation's shift	$14\,\mathrm{mV}$	[1,2,3]
K_{Ab}^{sNHE}	Hill parameter for sNHE's $\mathrm{HCO_3^-}_{\mathrm{i}}$ activation	$3\,\mathrm{mM}$	free parameter
K_{Ab}^{SLO3}	Hill parameter for SLO3's $\mathrm{HCO_3^-}_{\mathrm{i}}$ activation	$5\,\mathrm{mM}$	free parameter
$K_{\mathrm{A}}^{\mathrm{SLO3}}$, q	Hill parameters for SLO3's $\mathrm{pH_i}$ activation	$32\,\mathrm{nM}$, 1.65	[4]
α	relative increase of g_{SLO3} when phosphorylated	8.3	[8,9]
$\tilde{g}_{\mathrm{SLO3}}^{\mathrm{max}}$	max unphosphorylated SLO3's conductance	$6.4\,\mathrm{nS}$	[5]
V_{50}^{SLO3} , s_{SLO3}	SLO3's Boltzmann fit parameters	$12.8\,\mathrm{mV}$, $76\,\mathrm{mV}$	[5]
g_{l} , V_{leak}	leak current parameters	$1\,\mathrm{nS}$, $-40\,\mathrm{mV}$	[8,9]
k_{bicar} , $K_{\mathrm{A}}^{\mathrm{bicar}}$	Hill parameters for $\mathrm{HCO_3^-}$ transport	$\frac{1}{3}\,\mathrm{s^{-1}}$, $1\,\mu\mathrm{M}$	[11]*
$\mathrm{B_I}$	total intrinsic protons buffer	10^4	[11]*
$\mathrm{P_H}$	membrane's permeability to protons	$0.005\,\mathrm{cm\,s^{-1}}$	[12]*
C_{m} , S	sperm membrane's capacity and surface	$2.5\,\mathrm{pF}$, $250\,\mu\mathrm{m}^2$	[6]
V	sperm's cytosolic volume	$30\,\mathrm{fL}$	[7]
K_h	$\mathrm{CO_2}$'s hydration equilibrium constant	0.003	[10]

1.2 Figures

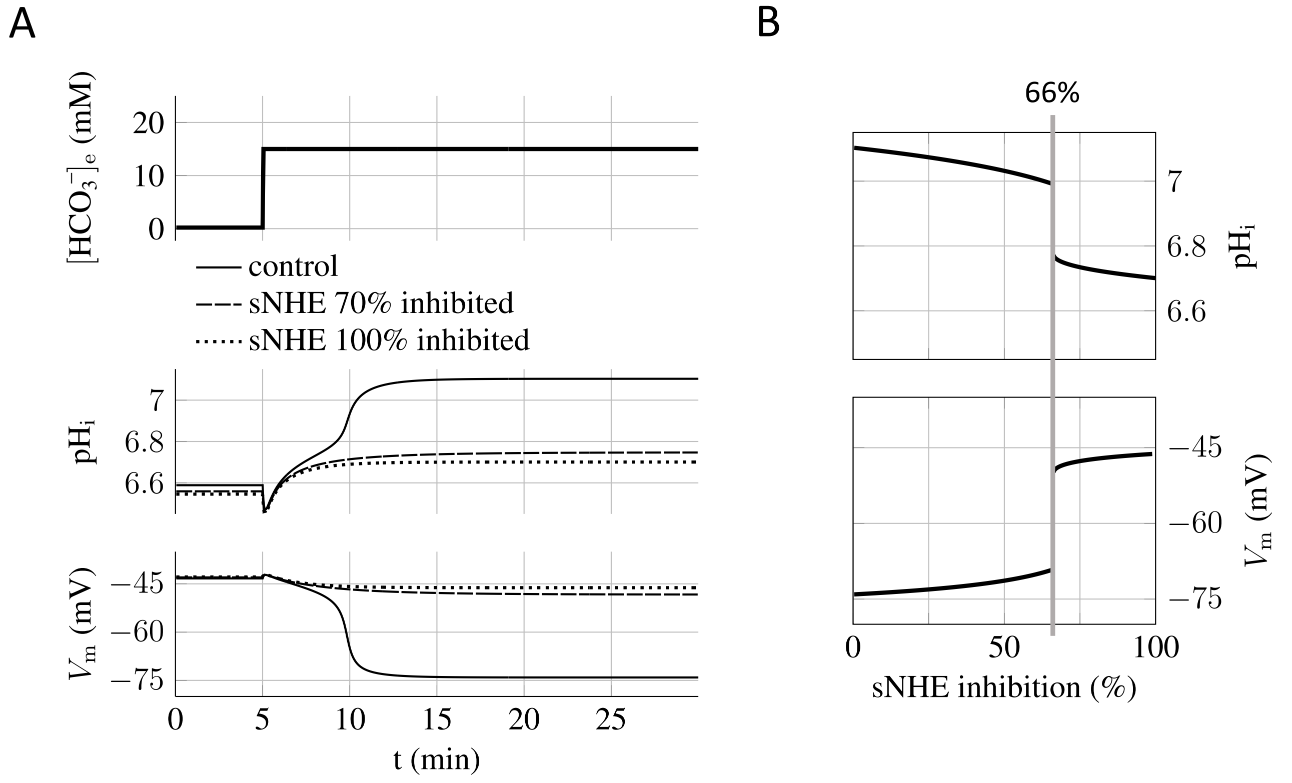

Figure S1. Capacitation dependence on sNHE inhibition.
(A) When sNHE is 70% inhibited, the transition to the capacitated state does not occur and the state is blocked at the values of $pH_i \simeq 6.8$ and $V_m \simeq$-50mV.
(B) Graph of the steady states of the sperm against the percentage of sNHE inhibition. These are the steady states reached after the incubation in capacitating medium (15mM bicarbonate) of spermatozoa previously prepared at 0.2mM bicarbonate. A threshold appears at 66% inhibition above which both the increases in pH_i and hyperpolarization are much reduced.
